# Pharmacological effects and phytochemical profile of methanolic *Odontosoria biflora* (Kaulf.) C.Chr. [Lindsaeaceae] extract in *Caenorhabditis elegans* models of Parkinson’s disease

**DOI:** 10.3389/fphar.2025.1662877

**Published:** 2026-01-21

**Authors:** Meysam Hamel Darbandi, Leslie Michelle M. Dalmacio, Jose Ma M. Angeles

**Affiliations:** 1 Department of Biochemistry and Molecular Biology, College of Medicine, University of the Philippines Manila, Manila, Philippines; 2 Department of Biochemistry, Nutrition and Molecular Biology, School of Medicine, Bohol Island State University, Tagbilaran, Bohol, Philippines; 3 Department of Parasitology, College of Public Health, University of the Philippines Manila, Manila, Philippines

**Keywords:** Odontosoria biflora, Parkinson’s disease, *Caenorhabditis elegans*, α-synuclein, dopaminergic neurons, locomotion, antioxidant activity, phytochemical profiling

## Abstract

Parkinson’s disease (PD) is a neurodegenerative disorder characterized by progressive dopaminergic neuronal loss, with oxidative stress and inflammation as key contributors to its pathogenesis. *Odontosoria biflora* (Kaulf.) C.Chr. [Lindsaeaceae], an endemic fern from Batanes Island, Philippines, is traditionally consumed as “tubho tea” and culturally associated with longevity. This study evaluated the pharmacological potential of *O. biflora* extract (OBE) in *Caenorhabditis elegans* models of PD. Leaves and stems were sequentially extracted using hexane (HOBE), ethyl acetate (EOBE), methanol (MOBE), and aqueous (AOBE) solvents. *C. elegans* N2, UA57, and NL5901 strains were cultured under standard conditions, and sublethal toxicity screening was conducted. The initial assay determined the effects of the four OBEs on dopaminergic neuronal loss in transgenic *C. elegans*, identifying MOBE as the most pronounced extract. MOBE was subsequently evaluated for α-synuclein aggregation, lifespan, mechanosensation, and locomotion. Antioxidant capacity was assessed using DPPH, ABTS, and FRAP assays as analytical tools, total phenolic content was determined, and phytochemical analysis was performed using high-resolution ultraperformance liquid chromatography coupled with electrospray ionization/quadrupole time-of-flight mass spectrometry. MOBE significantly reduced dopaminergic neuronal loss, decreased α-synuclein aggregation, extended lifespan, and improved mechanosensation and locomotion in transgenic *Caenorhabditis elegans* compared with both the negative and positive controls. Antioxidant assays demonstrated strong radical-scavenging activity consistent with its phenolic content (22.3 mg gallic acid equivalents [GAE]/g), and multiple metabolites were identified, including 1,4-dihydroxyanthraquinone, flavonoid 8-C glycosides, 2-O-rhamnosylvitexin, khellin, isovitexin, apigenin-8-C glucoside, benzoic acid, and pterosin G. Taken together, these findings suggest that MOBE exhibits pharmacological potential in *C. elegans* PD models and warrants further investigation in mammalian systems.

## 1 Introduction

Parkinson’s disease (PD) is the second most common age-related neurodegenerative disorder, affecting approximately 8.5 million people worldwide as of 2022, with prevalence expected to rise due to aging populations (Zafar and Yaddanapudi, 2023; Simon et al., 2020; Dorsey et al., 2018; Nabizadeh et al., 2024). The onset typically occurs between ages 55 and 65, with a lifetime risk of 2% for women and 3% for men (Dorsey et al., 2018).

Most PD cases (85%–90%) are idiopathic, while a minority are linked to genetic mutations (Ye et al., 2023). Environmental exposures are considered more relevant in late-onset cases, whereas genetic factors are thought to predominate in early-onset PD. Oxidative stress is a key contributor to neurodegeneration, and alpha-synuclein deposition in the basal ganglia is a hallmark pathological feature (Liu et al., 2017; Kouli et al., 2018; Trist et al., 2019). PD pathogenesis involves a combination of oxidative stress, inflammation, excitotoxicity, mitochondrial dysfunction, and protein aggregation (Olanow, 2007).

Although pharmacological treatments, such as levodopa and carbidopa, are available, there are currently no approved disease-modifying therapies (Gouda et al., 2022). Long-term levodopa therapy can cause motor complications, with 36.11% of Filipino PD patients developing levodopa-induced dyskinesia (Hughes et al., 2022; Stocchi et al., 2007). This highlights the need for effective screening models to identify novel therapeutic candidates.

*Caenorhabditis elegans* is a well-established PD model due to its genetic homology with humans, short lifecycle, and ethical advantages (Hughes et al., 2022; Kaletta and Hengartner, 2006; Brenner, 1974; Venkatesan et al., 2020). It can recapitulate PD-associated phenotypes, making it valuable for drug discovery (Cooper and Van Raamsdonk, 2018; Harrington et al., 2010). In this study, transgenic *Caenorhabditis elegans* strains UA57 and NL5901 were used to assess dopaminergic neuronal degeneration and alpha-synuclein aggregation.

UA57 worms overexpress the *cat-2* gene, encoding tyrosine hydroxylase, the rate-limiting enzyme in dopamine synthesis (Sulston and Horvitz, 1977; Omura et al., 2012; Jadiya and Nazir, 2012; Masoudi et al., 2014). Overexpression leads to excessive dopamine production and its conversion to 3,4-dihydroxyphenylacetaldehyde (DOPAL), which can generate free radicals, resulting in age-dependent degeneration of dopaminergic neurons (Harrington et al., 2010b; Masato et al., 2019). NL5901 worms express human alpha-synuclein fused to yellow fluorescent protein (YFP) under the control of the *unc-54* promoter, which drives robust expression in large body wall muscle cells, allowing for clear visualization of alpha-synuclein aggregates (Jadiya and Nazir, 2012; Fatima et al., 2014).

The NL5901 model has been widely used to screen for metabolites with potential anti-PD activity and to identify PD-related modifier genes (Long et al., 2022; Das et al., 2022). Natural products, particularly those with antioxidant and anti-inflammatory properties (Apak et al., 2013), have demonstrated neuroprotective potential (Amin et al., 2021; Balakrishnan et al., 2021). *Odontosoria biflora* (Kaulf.) C.Chr. [Lindsaeaceae], an endemic fern in Batanes traditionally consumed as tubho tea, is associated with the longevity of the Ivatan people (Zhou et al., 2017; Johnson et al., 2020; Amit and Sharma, 2020). However, its pharmacological effects in neurodegenerative disease models have not yet been studied. This preliminary investigation evaluates *Odontosoria biflora* extracts for their potential to mitigate PD-related phenotypes and extend lifespan in *C. elegans* models.

## 2 Materials and methods

### 2.1 Preparation of *Odontosoria biflora* extraction

The dried stems and leaves of *O. biflora* (Kaulf.) C.Chr. [Lindsaeaceae] were cut, rinsed with distilled water, and air-dried for 2 days. Taxonomic identification was confirmed at the Herbarium of the Institute of Biology, University of the Philippines Diliman. The plant material was cut into strips, soaked, and stored in an airtight container until it was ready for extraction. Four solvents, namely, hexane, ethyl acetate, methanol, and an aqueous solution, were used sequentially, progressing from nonpolar to polar. The procedure followed the cold maceration method (Dharajiya et al., 2017), with modifications that included soaking at room temperature, collecting the coffee cake, and drying it at the same temperature. The filtrates were subsequently separated, and the solvents evaporated using a rotary evaporator at 37 °C. For the aqueous fraction, approximately 1 L of filtrate was obtained, aliquoted into 40 mL per plate, and subjected to a two-step freezing process: initial freezing at 4 °C for 1 h, followed by storage at −80 °C for 24–72 h prior to lyophilization. After lyophilization, approximately 1 g of dried aqueous extract was recovered. The resulting dried extracts yielded approximately 28 g of hexane extract, 10 g of ethyl acetate extract, 4 g of methanol extract, and ∼1 g of aqueous extract. Each dried extract was stored at 4 °C until use. The dried extracts (hexane, ethyl acetate, methanol, and aqueous fractions) were reconstituted in 0.5% DMSO to prepare stock solutions and further diluted to the required working concentrations prior to mixing with *Escherichia coli* OP50. No sterile filtration step was applied, as extracts were co-administered with live OP50.

### 2.2 Sourcing and preparation of *Caenorhabditis elegans*

The *C. elegans* strains used in this study included N2 wild type, UA57 (dat-1pgfp + dat-1pcat-2), and NL5901 (unc-54palpha-synucleinyfp). All strains were obtained from the *Caenorhabditis* Genetics Center (CGC), University of Minnesota, Minneapolis, MN, USA. The wild-type and transgenic strains were cultivated and maintained at 20 °C on nematode growth medium (NGM) agar plates prepared according to standard protocols (Porta-de-la-Riva et al., 2012), which were seeded with non-pathogenic *E. coli* OP50 as a food source. Temperature-sensitive mutants were also maintained at 20 °C. For each treatment group, 15 worms at the L4 to young adult stage were manually transferred onto individual plates. Because *C. elegans* is a free-living, non-parasitic nematode and *E. coli* OP50 is a non-pathogenic bacterial strain (Manalo and Medina, 2018), all experimental procedures were conducted in compliance with Biosafety Level 1 (BSL-1) guidelines.

### 2.3 Age synchronization of *Caenorhabditis elegans*

Young adult worms were collected immediately after molting and transferred to fresh NGM plates seeded with *E. coli* OP50 to obtain populations of uniform age and developmental stage. After 1 hour of egg production, eggs were transferred to fresh NGM plates containing *E. coli* OP50. The emergence of synchronized larvae from the eggs was anticipated, and worms were transferred to new plates daily and maintained at 20 °C.

### 2.4 Modified bleaching method

Age synchronization of *C. elegans* was achieved using a modified bleaching method (Sulston and Horvitz, 1977). Freshly prepared M9 buffer was used to wash gravid adult worms from NGM plates. The worm suspension was vortexed for 2 min, pelleted, and then resuspended in bleaching solution, followed by another 1 min vortex. The suspension was then washed three to four times with M9 buffer until the solution was clear. Eggs were incubated overnight at 20 °C to allow for hatching. The resulting L1 larvae were inoculated onto NGM plates seeded with *E. coli* OP50 and maintained at 20 °C. After 48 h, L4 larvae were collected, followed by young adults after an additional 36 h.

### 2.5 Sublethal toxicity assay

The sublethal toxicity assay was performed to evaluate the effects of *O. biflora* extracts (OBEs) on the survival and general fitness of *C. elegans*. Age-synchronized L4-stage N2 wild type, UA57, and NL5901 worms were transferred to NGM plates and treated with OBEs at concentrations of 5, 10, 50, and 100 mg/mL dissolved in 0.5% DMSO (Jiang et al., 2017; Alokda and Van Raamsdonk, 2022). Worms were transferred to fresh NGM plates every other day, and the appropriate concentration of OBE was mixed with *E. coli* OP50 during feeding. Survival was recorded every 12 h for 3 days. Worms not treated with OBE served as the negative control. Each treatment group consisted of 15 worms, with three independent trials (n = 45 biological replicates).

### 2.6 Determination of the effects of four OBEs on dopaminergic neuronal loss

The UA57 (*dat-1p::gfp + dat-1p::cat-2*) *C. elegans* mutant strain was used to evaluate dopaminergic neuronal degeneration following exposure to four extracts of *O. biflora*: HOBE, EOBE, MOBE, and AOBE. Each extract was reconstituted in 0.5% DMSO and applied at a sublethal concentration of 5 mg/mL for at least 2 h at 20 °C until the worms reached the L4 stage (Manalo and Medina, 2018). After exposure, worms were washed three times with M9 buffer, transferred to fresh nematode growth medium (NGM) plates, and anesthetized using 20 mM sodium azide. Only the head region, specifically the four cephalic and two anterior deirid dopaminergic neurons, was observed using a fluorescence microscope (Evos® FL) every 24 h for three consecutive days. A minimum of *n* = 45 biological replicates was used, comprising three independent trials with 15 worms per group. Fluorescent images were analyzed using ImageJ v1.8.0_172 (National Institute of Health, NIH, Bethesda, MD, United States). The proportion of intact neurons was calculated along with the minimum, maximum, and average green fluorescent protein (GFP) intensity per neuron (Manalo and Medina, 2018). Results were reported as mean ± standard error of the mean (SEM). Neurons were classified as lost if GFP fluorescence was absent or if small, rounded fluorescent bodies were observed (Manalo and Medina, 2018). The overall corrected total cell fluorescence (CTCF) was calculated for 45 biological replicates (three independent trials, each with 15 worms). The variance was determined using the standard error of the means, and values at day 3 were normalized to baseline CTCF values at day 0 using the following formula:

$$\text{Overall CTCF after day }3=\frac{\text{mean}\;\text{CTCF}\;\left(\text{day}\;3\right)}{\text{mean}\;\text{CTCF}\;\left(\text{day}\;0\right)\;}$$

The overall CTCF at day 3 was normalized using the baseline CTCF of the sample at day 0. The 1 mM DOPA (L-DOPA ethyl ester, SML0091, Sigma-Aldrich, USA) served as the positive control, while *E. coli* OP50-fed worms served as the negative control.

Following the sublethality assay, a two-stage experimental design was implemented. In the initial stage, four *O. biflora* solvent extracts (HOBE, EOBE, MOBE, AOBE) were assessed at a single sublethal concentration (5 mg/mL in 0.5% DMSO) to determine which extract most effectively preserved dopaminergic neurons in UA57. The concentration of 5 mg/mL was selected as it was the highest dose that maintained ≥90% survival at 72 h across all strains and solvents, thereby ensuring maximal sensitivity for detecting neuroprotective effects while remaining within non-lethal limits. A dose–response assessment was not conducted at this stage, as the objective was to identify the extract exhibiting the most potent pharmacological neuroprotective activity rather than to estimate potency.

In the subsequent stage, the extract demonstrating the most pronounced effect was advanced to additional assays, including lifespan and α-synuclein misfolded protein assay, using three sublethal concentrations (0.05, 0.5, and 5 mg/mL) to determine the concentration range associated with pharmacological activity.

### 2.7 Determination of the effect on α-synuclein misfolded protein of the selected *Odontosoria biflora* extract

To determine whether the selected extract of *O. biflora* could ameliorate Parkinson’s disease (PD)-related pathology, an established transgenic *C. elegans* model (NL5901) expressing human α-synuclein fused to yellow fluorescent protein (YFP) was used following the standard protocol (Hughes et al., 2022). Age-synchronized eggs were transferred to nematode growth medium (NGM) plates, fed with or without selected extract (reconstituted in 0.5% DMSO), and incubated at 20 °C until they reached the L4 stage. Worms were then washed three times with M9 buffer, transferred to fresh NGM plates, and anesthetized using 20 mM sodium azide. Anesthetized worms were mounted and observed under a fluorescence microscope to visualize the α-synuclein misfolded protein in the body wall muscle. Quantification of misfolded protein was performed using ImageJ v1.51w through a semi-automated process. For consistency, the same anatomical region of the body wall muscle near the head was analyzed in each worm. Images were scaled, the background was removed, and they were processed using the watershed function to separate overlapping particles. ImageJ was used to calculate both the number of aggregates per image and the mean aggregate size in calibrated units (µm^2^). The resulting data were imported into GraphPad Prism v10 for statistical analysis, with results expressed as mean ± standard error of the mean (SEM) and visualized with appropriate error bars (Hughes et al., 2022).

### 2.8 Determination of the effect of the selected *Odontosoria biflora* extract on the lifespan of *Caenorhabditis elegans*

The lifespan assay was conducted using age-synchronized *C. elegans* obtained via the modified bleaching method. Wild-type N2 and NL5901 strains were treated with selected *O. biflora* extract for the test groups, 1 mM DOPA as a positive control, and OP50 as a negative control. The survival status of each worm, categorized as alive, dead, or missing, was recorded daily until all worms had died (Masoudi et al., 2014). Worms were transferred to fresh nematode growth medium plates every other day to replenish the OP50 food source and prevent confounding from newly hatched progeny (Hughes et al., 2022). Worms were considered alive if they responded to a gentle touch with a platinum wire pick; absence of response was recorded as death. Survival data from all groups were used to generate Kaplan–Meier survival curves in GraphPad Prism version 9.2.0 (283), and results were expressed as mean lifespan ±standard error of the mean (SEM).

### 2.9 Determination of the effects of the selected *Odontosoria biflora* extract on mechanosensation and locomotion in a *Caenorhabditis elegans* model of Parkinson’s disease

#### 2.9.1 Mechanosensation assay

The mechanosensation assay, adapted from Goodman (2006), was performed on the *C. elegans* Parkinson’s disease model (UA57). The assay consisted of five tests: plate tap, hard touch, nose touch, gentle touch, and head touch. At least 45 worms were used for each treatment group. The positive control group received 1 mM DOPA, while the negative control group received no treatment and was fed only OP50. For the plate tap test, worms were transferred from the treatment plates to examination plates. The plates were tapped three to five times, and each worm was observed for backward reversal movement, indicating the presence of active dopaminergic neurons (Manalo and Medina, 2020). The head touch test was performed using a worm picker by pinching the mid-ventral region of the worm. This pinching stimulated nociceptor neurons and triggered backward movement (Manalo and Medina, 2020). A gentle touch test, comprising a head subtest, was performed. The head touch was performed using an eyelash hair and slowly moving the hair across the head (above the pharynx) of the worm. Head touch activates forward reversal movement on the *C. elegans* (Manalo and Medina, 2020). Quantification was performed using a binary scoring method, where a value of one was assigned for a positive response and 0 for a negative response. The scoring was based on observable behaviors with established response patterns in *C. elegans*, as previously reported by Chalfie et al. (2014). Worm movement was observed using a stereomicroscope equipped with an AMScope® camera connected to a laptop. The mechanosensation assay was conducted over 3 days, with five tests performed every 24 h. Graphical representations were generated using GraphPad Prism version 10.0.0.

#### 2.9.2 Locomotion assay

Worms were subjected to various treatments for at least 2 h before testing. Each worm was then individually transferred to a plain NGM examination plate to reduce the influence of external stimuli. They were allowed to move freely for 1 minute, based on evidence that *C. elegans* demonstrates an escape response for approximately the first 100 s and suppresses spontaneous reversals for up to 1 minute following light mechanical stimulation (Zhao et al., 2003). After this acclimation period, each worm was recorded for 20 s, during which specific locomotion behaviors were measured: short reversals, long reversals, total reversals, omega turns, and body bends (Zhao et al., 2003; Hart, 2006). Following observation, worms were transferred back to individually labeled OP50-seeded NGM plates for continued assays up to day three. Behavioral responses were recorded as absolute counts (i.e., number of responses per 20 s). Graphical representations were generated using GraphPad Prism version 10.0.0.

### 2.10 Determination of the chemical composition of the selected *Odontosoria biflora* extract

#### 2.10.1 Analytical determination of radical-scavenging and reducing properties of the selected *Odontosoria biflora* extract

Chemical assays were performed solely to characterize the radical-scavenging and reducing properties of the selected *O. biflora* extract. These analytical methods provide no direct evidence of pharmacological activity or therapeutic potential.

#### 2.10.2 DPPH assay

The 2,2-diphenyl-1-picrylhydrazyl (DPPH) assay was used to quantify the radical-scavenging capacity of the extract (Ribeiro et al., 2008; Bueno and Yu, 2021). DPPH is a stable free radical with a characteristic purple color (maximum absorbance at 517 nm), which becomes yellow upon reduction by sample metabolites. Briefly, 1 mL of diluted *O. biflora* extract (100, 150, 200, and 250 µg gallic acid equivalents [GAE]/mL) was mixed with 5 mL of 0.1 mM DPPH in methanol and incubated at room temperature in the dark for 20 min. Absorbance was then measured at 517 nm against a blank consisting of distilled water. The DPPH reagent in water served as the control. Radical-scavenging capacity (%) was calculated as:

$$\text{Inhibition }\%=[(\text{Absorbance of Control}-\text{Absorbance of Sample }\left(\text{OBE}\right)]\;/\text{ Absorbance of Control}]\times 100$$

#### 2.10.3 ABTS assay

The 2,2′-azino-bis (3-ethylbenzothiazoline-6-sulfonic acid) (ABTS) assay was used to determine the radical-scavenging capacity of the extract (Rufino et al., 2010; Re et al., 1999). In this method, ABTS is converted to its radical cation (ABTS^+^•) by oxidation with sodium persulfate, producing a blue-green solution with peak absorbance at 734 nm. Antioxidant metabolites in the extract reduce ABTS^+^• to its colorless form, and the decrease in absorbance is proportional to radical-scavenging capacity. Briefly, 0.01 mL of diluted *O. biflora* extract (190, 380, 560, and 750 ng GAE per 0.01 mL) was added to 3 mL of freshly prepared ABTS reagent solution, vortex-mixed, and incubated at room temperature for 5 min. Absorbance was measured at 734 nm, using distilled water to calibrate the instrument to zero. The reagent solution containing 0.01 mL of 90% methanol served as the control. Radical-scavenging activity (%) was calculated as:

$$\text{Inhibition }\%=[(\text{Absorbance of Control}-\text{Absorbance of Sample }\left(\text{OBE}\right)]/\text{ Absorbance of Control}]\times 100$$

#### 2.10.4 FRAP assay

The Ferric Reducing Antioxidant Power (FRAP) assay was conducted to evaluate the electron-donating capacity of the selected *O. biflora* extract, reflecting its reducing potential (Ribeiro et al., 2008). This assay measures the reduction of ferricyanide [Fe(CN)_6_]^3-^ to ferrocyanide [Fe(CN)_6_]^4-^, which then reacts with ferric ions (Fe^3+^) to form Prussian blue (Fe_4_ [Fe(CN)_6_]_3_). The intensity of the resulting blue color, measured at 700 nm, is directly proportional to reducing power.

Briefly, 1.0 mL of extract was mixed with 1.0 mL of 0.2 M phosphate buffer (pH 6.6) and 1.5 mL of 1% (w/v) potassium ferricyanide. After incubation at 50 °C for 30 min, the reaction was stopped by adding 1.5 mL of 10% (w/v) trichloroacetic acid. Aliquots of 2.0 mL were then combined with 2.0 mL distilled water and 0.5 mL of 0.1% (w/v) ferric chloride. Absorbance was measured at 700 nm against the reagent blank. Ascorbic acid was used as the positive control.

#### 2.10.5 Total phenolic content

The total phenolic content of the selected *O. biflora* extract was determined using the Folin–Ciocalteu method with gallic acid as the standard. A calibration curve was prepared using gallic acid standard solutions ranging from 100 to 600 ppm. For the assay, 0.5 mL of selected *O. biflora* extract was mixed with 10.0 mL of distilled water, 1.0 mL of Folin–Ciocalteu reagent, and 3.0 mL of sodium carbonate. The mixture was diluted to a final volume of 25.0 mL, heated at 50 °C for 5 min, then cooled to room temperature for 30–60 min. The absorbance was measured at 765 nm using a UV–Vis spectrophotometer, with distilled water as the blank. Total phenolic content was calculated from the linear regression equation of the gallic acid calibration curve and expressed as milligrams of gallic acid equivalents (GAE) per 100 g of dry matter (Genwali et al., 2013). All reagents were sourced from the National Institute of Molecular Biology and Biotechnology, University of the Philippines, Los Baños.

#### 2.10.6 Metabolite profiling

About 5 mg of selected *O. biflora* extract was prepared and diluted accordingly based on the extract’s solubility. The selected *O. biflora* extract was diluted directly with 2 mL LC-MS grade methanol. Afterwards, the samples were filtered using a 0.2 µm PTFE syringe filter into clear LC-MS vials. Water, methanol, and methanol with DMSO solvents were used as a blank for the analysis, while quercetin was used as the standard for system suitability. UPLC-ESI-QTOF-MS analysis. Metabolite profile screening was performed on a Waters ACQUITY I-Class UPLC coupled to a Waters Xevo G2-S QTOF mass spectrometer. A reverse-phase Waters ACQUITY HSS C18 column (2.1-mm internal diameter × 100-mm length, 1.8-μm particle size) was used. The mass analyzers were calibrated using a 0.5 mM sodium formate solution to enhance the instrument’s mass accuracy. A 200-pg/µL leucine-enkephalin in 50:50 (v/v) acetonitrile–water +0.1% formic acid solution was used as the lock mass (m/z 556.2771), sprayed at an interval of 30 s throughout the LC run time, and scanned for 1.5 s each spray. The mobile phases consisted of (A) ultrapure water with 0.1% formic acid, and (B) acetonitrile with 0.1% formic acid. A gradient elution was as follows: 15%–40% B (0–1.67 min), 40%–55% B (1.67–5.00 min), 55%–75% B (5.00–6.67 min), 75%–80% B (6.67–10.84 min), 80%–95% B (10.84–13.34 min), 95% to 15% B (13.34–15.01 min), and 15% to 5% B (15.01–18.00 min). The LC-MS data were acquired using the Masslynx 4.2 software. The mass range was from 50 to 1,500 Da, 40 V for cone voltage, 80 V for source offset, 3.0 kV for capillary voltage, 120 °C for source temperature, and the desolvation temperature was at 450 °C. The desolvation gas (nitrogen) and the cone gas (argon) flow rates were set at 600 L/h and 100 L/h, respectively. The column temperature used was 30 °C, while the sample temperature was set at 15 °C. Electrospray ionization was performed in the positive ionization mode, and data-independent acquisition mode (MSE mode) in continuum format was utilized, with a low collision energy of 6 eV and a ramp from 30 to 50 eV for high collision energy scans (Clauser et al., 1999). The detector for the PDA was set to range from 190 to 500 nm. The injection volume will be 2 μL, with a flow rate of 0.25 mL/min. Analysis. All the reagents used were from Pascual Pharma Corp Laboratory (Philippines).

### 2.11 Statistical analysis

All functional assays used a minimum of number of biological replicates (n = 45) consisting of three independent trials with 15 worms per group. In the sublethality assay, the Kaplan-Meier analysis was performed, and all results were expressed as the mean ± standard deviation. One-way analysis of variance (ANOVA) and pairwise t-test with Hochberg correction were performed for dopaminergic neuronal loss assay, alpha synuclein assay and lifespan assay. The results of these assays were expressed as the mean ± SEM. The antioxidant assays used two-way ANOVA followed by Sidak multiple comparison tests. Data values were expressed as the mean ± SD. For the mechanosensation and locomotion assays, two-way ANOVA followed by post-hoc Tukey multiple comparison test was used. Data are presented as the mean ± SEM.

## 3 Result

### 3.1 Sublethal concentrations of O. biflora extracts (OBEs) on *Caenorhabditis elegans*

The maximum tolerable concentration of *O. biflora* extracts (OBEs) was evaluated in 3 *C. elegans* strains UA57, NL5901, and N2 using a sublethal toxicity assay as a preliminary assessment. A sublethal concentration was defined as the dose at which ≥90% of the population survived within 72 h, ensuring sufficient sample size for statistical analysis even at the survival threshold.

**FIGURE 1:**
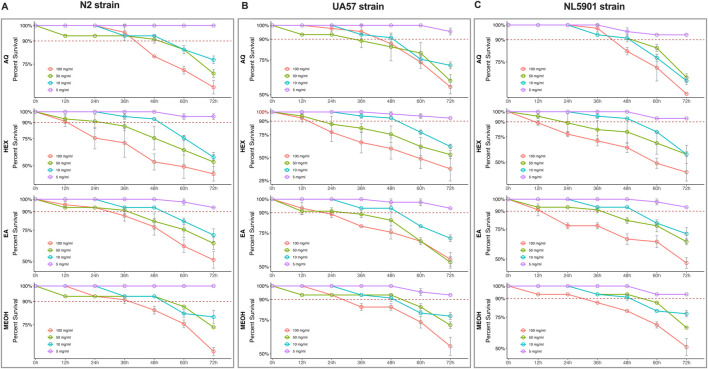
Survival of *Caenorhabditis elegans* strains **(A)** N2, **(B)** UA57, and **(C)** NL5901 following exposure to *Odontosoria biflora* extracts at concentrations of 5, 10, 50, and 100 mg/mL over a 72-h period. A minimum of *n* = 45 biological replicates was used, consisting of three independent trials with 15 worms per group. Kaplan-Meier analysis was performed, and all results are expressed as the mean ± standard deviation.

Four concentrations (5, 10, 50, and 100 mg/mL) of each solvent extract, hexane (HOBE), ethyl acetate (EOBE), methanol (MOBE), and aqueous (AOBE), were tested. Across all strains, 5 mg/mL consistently resulted in >90% survival (Figure 1: panel A, strain N2; panel B, strain UA57; panel C, strain NL5901). In contrast, higher concentrations reduced survival below 90%, limiting the number of viable samples for robust statistical evaluation.

For subsequent pharmacological assays, two additional sublethal dilutions were prepared from the 5 mg/mL stock: 0.5 mg/mL (10^−1^) and 0.05 mg/mL (10^−2^). Thus, all downstream experiments were conducted at 0.05, 0.5, and 5 mg/mL. Each assay utilized a minimum of *n* = 45 biological replicates, comprising three independent trials with 15 worms per group. Survival data were analyzed using Kaplan–Meier survival curves, and results are presented as mean ± standard deviation (SD).

### 3.2 Effects of OBEs on dopaminergic neuronal loss

To evaluate the neuroprotective pharmacological effects of *O. biflora* prepared with Hexane (HOBE), ethyl acetate (EOBE), methanol (MOBE), and aqueous (AOBE), the *C. elegans* strain UA57 was utilized. This transgenic strain overexpresses tyrosine hydroxylase, leading to excessive dopamine production, which results in dopamine-induced neurodegeneration and DOPAL accumulation, promoting oxidative stress and dopaminergic neuron degeneration (Trist et al., 2019; Pingale and Gupta, 2021; Plotegher and Duchen, 2017). This model enabled the identification of the pharmacologically active extract for subsequent assays. Neurodegeneration was assessed by measuring the green fluorescent protein (GFP) intensity in dopaminergic neurons using a fluorescence microscope over 72 h, where a decrease in intensity from baseline indicated neurodegeneration.

**FIGURE 2:**
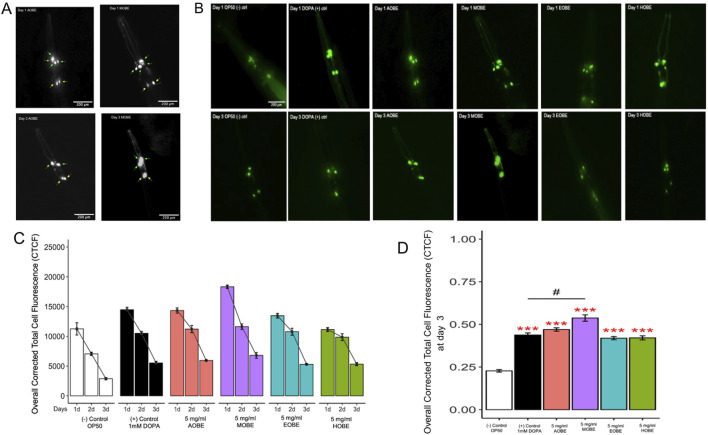
Effect of *O.biflora extracts* on Transgenic *Caenorhabditis elegans* UA57 in the dopaminergic neuronal loss assay. MOBE protects dopaminergic (DA) neurons. **(A)** Representative grayscale fluorescent images of *Caenorhabditis elegans* dopaminergic neurons at 200 µm magnification. Four cephalic (CEP) neurons are indicated by green arrows and two anterior deirid (ADE) neurons by yellow arrows. Images were captured on Day 1 and Day 3 following treatment with aqueous (AOBE) and methanolic (MOBE) extracts of *Odontosoria biflora* (Kaulf.) **(C)**Chr. [Lindsaeaceae]. By Day 3, the AOBE-treated group showed reduced fluorescence intensity and dopaminergic neuronal loss, with only two CEP neurons remaining. In contrast, the MOBE-treated group demonstrated greater neuronal preservation, with all four CEP neurons retained, though some appear overlapped. **(B)** Transgenic *C. elegans* UA57 with cat-2 overexpression and GFP-labeled dopaminergic neurons were treated with OP50 (negative control), DOPA (1 mM; positive control), AOBE (5 mg/mL), MOBE (5 mg/mL), EOBE (5 mg/mL), and HOBE (5 mg/mL), and monitored over 3 days using fluorescence microscopy. **(C)** Images were analyzed using ImageJ. **(D)** MOBE significantly protected DA neurons over 3 days post-adulthood, while DOPA, as the positive control, showed limited neuroprotection. A minimum of n = 45 biological replicates was used, consisting of three independent trials with 15 worms per group. One-way analysis of variance (ANOVA) and pairwise t-tests with Hochberg correction were performed. Results are expressed as mean ± SEM. Hash marks (###) indicate a significant difference compared to the positive control (p ≤ 0.0001). Asterisks (***) indicate a significant difference compared to the negative control (p ≤ 0.0001).

Figures 2A,B show a general trend of initially high GFP intensity across all treatment groups on Day 1 compared to the negative control (OP50), followed by a gradual decline by Day 3. Figure 2A shows a grayscale fluorescent image of *C. elegans* dopaminergic neurons. On Day 1, all neurons appear intact in both groups. By Day 3, fluorescence intensity diminishes in both treatments; however, MOBE-treated worms retain more visible neurons than those treated with AOBE, suggesting better neuronal preservation. However, GFP intensity decreased in all groups over time, *C. elegans* treated with 5 mg/mL methanolic *O. biflora* extract (MOBE) exhibited significantly higher GFP intensity (p < 0.001) on day 3 compared to both the negative control and the positive control (1 mM DOPA) (Figure 2D).

Worms fed with OP50 as a negative control showed a progressive and significant decrease in GFP fluorescence over 3 days, indicating ongoing dopaminergic neurodegeneration. This serves as the baseline for degeneration in UA57 worms without any neuroprotective intervention. The lowest overall CTCF values were recorded in this group by day 3, confirming its role as the degenerative baseline (Figures 2C,D).

The DOPA 1 mM positive control slightly delayed the loss of dopaminergic neuron fluorescence by Day 1 compared to the negative control. However, by day 3, there was still a notable decrease in GFP intensity. Although statistically different from the OP50 group (p < 0.0001), the protective effect of DOPA was not as strong as observed in the methanolic extract treatments. This reflects DOPA’s partial neuroprotective effect, as it also suggests that dopamine is prone to both spontaneous and metal-catalyzed oxidation at its catechol group, forming reactive ortho-quinones (Masato et al., 2019). Enzymatic oxidation can also generate superoxide radicals, resulting in cellular damage. These dopamine-derived quinones can form neurotoxic metabolites, such as salsolinol, which disrupts catecholamine metabolism and induces oxidative stress and mitochondrial dysfunction (Trist et al., 2019).

Worms treated with AOBE showed a modest neuroprotective effect, with significantly higher GFP intensity than the positive and negative groups on day 3 (p < 0.001). This indicates that while AOBE slowed neurodegeneration, its effect was limited, possibly due to the lower solubility of polyphenols in water, as supported by prior phytochemical profiling studies.

As highlighted earlier, MOBE-treated worms displayed the highest retention of GFP fluorescence by Day 3, indicating the most potent neuroprotective effect among all extract types. The fluorescence levels were significantly higher than both negative and positive controls (p < 0.0001). This suggests that methanol effectively extracts polyphenols and neuroprotective metabolites from *O. biflora*, consistent with metabolite profiling, which shows that methanol extracts from *O. biflora* are richer in phenolic content.

As for EOBE and HOBE, both showed minimal protection compared to the positive control. The GFP intensity of the HOBE decreased significantly over 3 days, and by day 3, the values were marginally higher than those of OP50. This is in contrast with EOBE, which had substantially higher fluorescence on day 3 than OP50 (p < 0.001). Hexane’s limited ability to extract polar bioactive metabolites, such as polyphenols, could mediate the neuroprotective effect of the extract.

Treatment with 5 mg/mL MOBE significantly slowed dopaminergic neurodegeneration compared with the other extracts tested. The neuroprotective pharmacological effects of MOBE were demonstrated by sustained GFP fluorescence intensity in dopaminergic neurons over the 72-h observation period. Based on this, 5 mg/mL MOBE was selected for subsequent assays. A total of 45 biological replicates were analyzed, comprising three independent trials with 15 worms per group. Statistical evaluation of dopaminergic neuronal loss was performed using one-way analysis of variance (ANOVA), followed by pairwise t-tests with Hochberg correction. Results are expressed as mean ± standard error of the mean (SEM).

### 3.3 Effects of MOBE on α-synuclein misfolding protein

MOBE was selected based on its activity compared to the positive control and was tested consequently at concentrations of 0.05, 0.5, and 5 mg/mL using the *C. elegans* strain NL5901. This strain constitutively expresses human α-synuclein fused to yellow fluorescent protein (YFP) in the body wall muscle under the control of the unc-54 promoter. It is widely used as a pharmacological model of Parkinson’s disease (PD) because the progressive accumulation of α-synuclein misfolded protein in muscle cells mimics PD-related pathology and motor decline (Wong and Krainc, 2017; Van Ham et al., 2008; Long et al., 2022). Worms were exposed to MOBE from day 1 to day 7 of adulthood, and α-synuclein misfolded protein was visualized in the anterior head region of L4 worms. Both the number and size (µm^2^) of aggregates were quantified using ImageJ software, following the established methodology of Hughes et al. (2022). Untreated L4 worms typically exhibited 100–130 aggregates, each ranging from 0.23 to 2.9 µm^2^.

**FIGURE 3:**
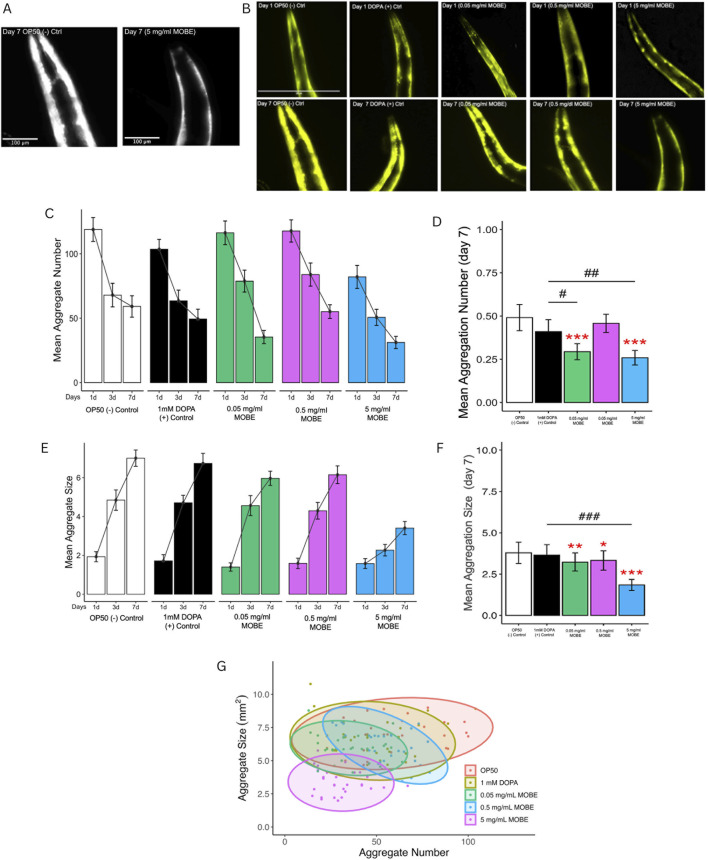
Effect of MOBE on α-synuclein misfolded protein (aggregation) in NL5901 *Caenorhabditis elegans*. Transgenic worms expressing human α-synucleinYFP in body wall muscles were treated with OP50 (negative control), DOPA (1 mM; positive control), and MOBE (5, 0.5, and 0.05 mg/mL), and monitored over 7 days by fluorescence microscopy. **(A)** Representative black-and-white fluorescent image of *Caenorhabditis elegans* NL5901 on Day 7 showing reduced α-synuclein misfolded protein in worms treated with MOBE compared to the OP50 control. **(B)** Representative images on day 1 and day 7 show visible misfolded protein. **(C–F)** Quantification reveals that MOBE at 5 mg/mL significantly reduced aggregate number and size compared to other doses, DOPA, and OP50. **(G)** Scatter plot on day 7 confirms the anti-aggregation effect of MOBE (5 mg/mL). A minimum of *n* = 45 biological replicates was used, consisting of three independent trials with 15 worms per group. One-way analysis of variance (ANOVA) and pairwise *t*-tests with Hochberg correction were performed. Results are expressed as mean ± SEM. Hash marks (###) indicate a significant difference compared to the positive control (p ≤ 0.0001). Asterisks (***) indicate a significant difference compared to the negative control (p ≤ 0.0001).

Figure 3A shows a representative fluorescent image of NL5901 worms, demonstrating that treatment with 5 mg/mL MOBE markedly reduced visible α-synuclein aggregates on day 7 compared to the negative control (OP50). On day 1, all groups exhibited similar numbers of misfolded proteins (91–121) with small aggregate sizes (0.23–2.9 µm^2^; Figures 3B–E). This pattern reflects dynamic aggregation, in which small aggregates act as “seeds” for growth and fusion into larger, more neurotoxic aggregates during aging (Vidović and Rikalović, 2022; Volpicelli-Daley et al., 2011).

By day 7, 5 mg/mL MOBE significantly reduced both the number and size of α-synuclein aggregates compared to controls (p < 0.001 and p < 0.01; Figures 3B,C). Although aggregate size increased across all groups with age, the 5 mg/mL MOBE treatment maintained a lower average size (1.84 µm^2^; Figures 3F,G). Larger aggregates are known to exacerbate oxidative stress, disrupt proteostasis, and impair mitochondrial function, thereby worsening neurotoxicity (Peelaerts et al., 2015; Burre et al., 2018).

Further analysis using scatter plots (Figure 3F) confirmed that the 5 mg/mL MOBE treatment significantly reduced both the number and size of aggregates compared to negative and positive controls (p < 0.001 and p < 0.01). Interestingly, the 0.05 mg/mL treatment showed fewer and smaller aggregates than the 0.5 mg/mL group (p < 0.001), suggesting a non-linear dose–response that is typical of plant-derived metabolites. At low doses (0.05 mg/mL), hormesis may occur, as mild stress activates protective pathways. In contrast, the reduced effect at 0.5 mg/mL may reflect antagonistic interactions among metabolites. Nonetheless, the 5 mg/mL MOBE consistently produced the most potent pharmacological effect, markedly reducing α-synuclein misfolded protein burden and slowing plaque development.

A minimum of 45 biological replicates was analyzed, consisting of three independent trials with 15 worms per group. Data were evaluated using one-way ANOVA followed by pairwise t-tests with Hochberg correction. Results are expressed as mean ± SEM. PERMANOVA was applied to aggregate number and size, as it accounts for multivariate dispersion across treatments.

### 3.4 Effect of MOBE on the lifespan of *Caenorhabditis elegans*

**FIGURE 4:**
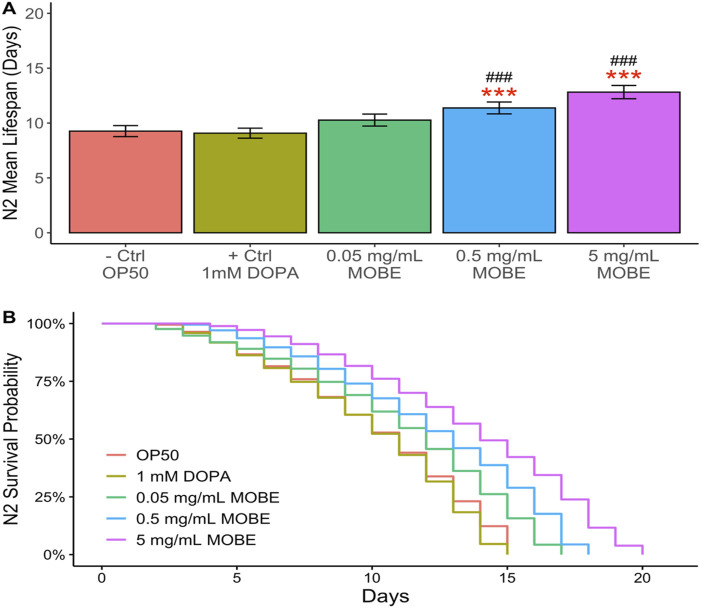
MOBE (5 mg/mL) significantly prolongs lifespan in N2 wild-type *Caenorhabditis elegans*. **(A)** Mean lifespan and **(B)** survival percentage of worms treated with MOBE at 0.05, 0.5, and 5 mg/mL. A minimum of *n* = 45 biological replicates was used, consisting of three independent trials with 15 worms per group. One-way ANOVA and pairwise t-tests with Hochberg correction were performed. Results are expressed as mean ± SEM. Hash marks (###) indicate a significant difference compared to the positive control (p ≤ 0.0001); asterisks (***) indicate a significant difference compared to the negative control (p ≤ 0.0001).

**FIGURE 5:**
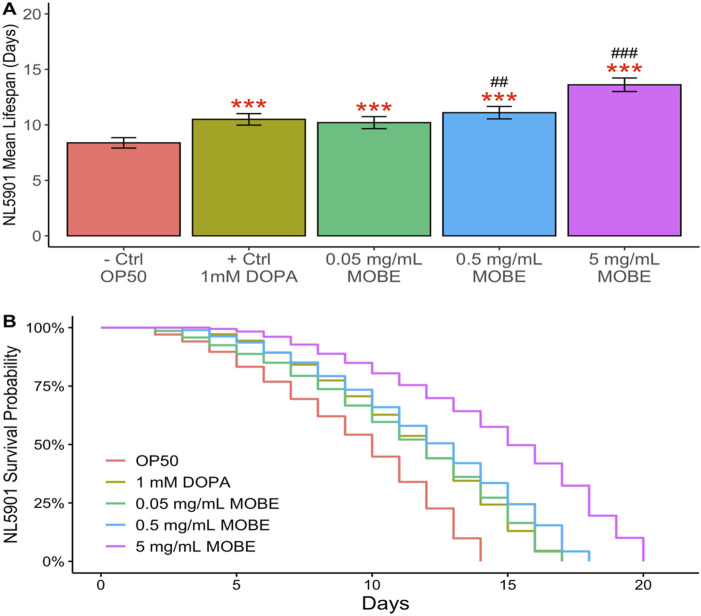
MOBE (5 mg/mL) significantly prolongs lifespan in NL5901 transgenic *Caenorhabditis elegans*. **(A)** Mean lifespan and **(B)** survival percentage of worms treated with MOBE at 0.05, 0.5, and 5 mg/mL. A minimum of *n* = 45 biological replicates was used, consisting of three independent trials with 15 worms per group. One-way ANOVA and pairwise t-tests with Hochberg correction were performed. Results are expressed as mean ± SEM. Hash marks (###) indicate a significant difference compared to the positive control (p ≤ 0.0001); asterisks (***) indicate a significant difference compared to the negative control (p ≤ 0.0001).

To evaluate the pharmacological effects of MOBE *in vivo*, lifespan assays were conducted using *C. elegans*. Both N2 wild-type and NL5901 strains were exposed to MOBE at concentrations of 0.05, 0.5, and 5 mg/mL starting from the L4 stage. A concentration-dependent trend was observed, with a significant extension of lifespan at 5 mg/mL (Figures 4, 5). At this concentration, MOBE increased the mean lifespan of N2 worms by 13.61% compared to 9.26% in the untreated control (Figure 4A), while NL5901 worms exhibited a 13.85% increase compared to 8.37% in the untreated control (Figure 5A). Survival analysis further showed that, at 50% survivability, MOBE-treated N2 and NL5901 worms survived until days 15 and 16, respectively, whereas the untreated controls declined earlier (Figures 4B, 5B) (Hughes et al., 2022). These findings demonstrate that MOBE exerts pharmacological effects *in vivo*, significantly prolonging lifespan in both wild-type and Parkinson’s disease model *C. elegans*.

### 3.5 Effect of MOBE on mechanosensation and locomotion

**FIGURE 6:**
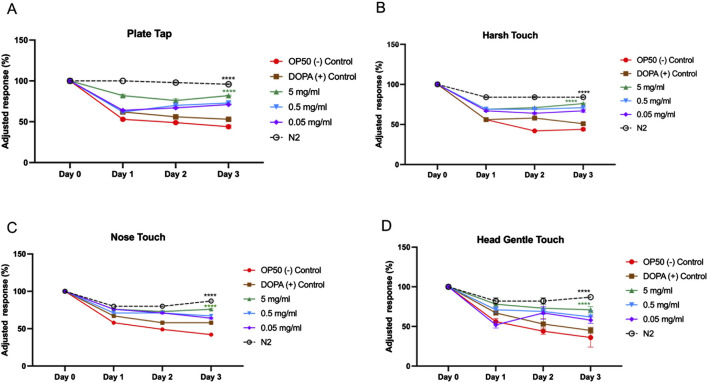
Mechanosensory response assay in UA57 *Caenorhabditis elegans* following treatment with MOBE. Representative results of mechanosensation assays **(A)** Plate tab, **(B)** Harsh touch, **(C)** Nose touch, **(D)** Gentle head touch in UA57 transgenic worms treated with 5 mg/mL, 0.5 mg/mL, and 0.05 mg/mL of MOBE, alongside negative (OP50) and positive (1 mM DOPA) controls. MOBE at 5 mg/mL showed the most significant preservation of mechanosensory responsiveness, particularly in harsh and gentle touch assays, indicating functional neuroprotection. Response decline was most rapid in DOPA-treated worms, consistent with dopaminergic overload. A minimum of n = 45 biological replicates was used, consisting of three independent trials with 15 worms per group. two-way ANOVA followed by post-hoc Tukey multiple comparison test was used. Bar graphs represent the average number of responding worms across trials. Error bars indicate standard error of the mean (SEM). p < 0.05 vs. OP50.

To functionally validate the neuroprotective potential of MOBE observed in the dopaminergic neuronal loss assay, locomotor activity and mechanosensory responses were assessed in the transgenic *C. elegans* strain UA57. Mechanosensory integrity was evaluated using tactile and vibratory stimuli, including plate tap, harsh touch, nose touch, and gentle head touch (Figures 6A–D). In the negative control (OP50-fed worms), responses declined sharply by Day 1 and remained low through Day 3. DOPA-treated UA57 exhibited comparable or slightly greater declines.

MOBE at 5 mg/mL significantly attenuated this sensory decline. For plate tap and harsh touch assays, MOBE-treated animals maintained higher adjusted response percentages throughout the experiment (p < 0.0001 vs. OP50, Figures 6A,B). Nose touch and gentle head touch responses were also significantly preserved (Figures 6C,D).

**FIGURE 7:**
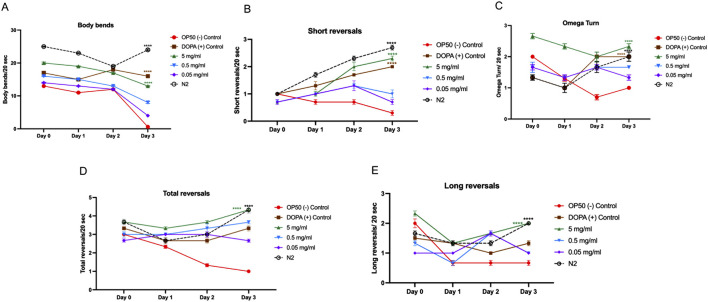
Locomotion behavior of UA57 *Caenorhabditis elegans* following MOBE treatment across multiple motor parameters. **(A)** Body bends significantly decreased in OP50-treated worms, while MOBE (5 mg/mL) preserved normal bending., **(B)** Short reversal increased in MOBE and DOPA-treated worms, but remained low in the negative control. **(C)** omega turn were maintained or improved with MOBE treatment, in contrast to the decline seen in OP50 control. **(D)** Total reversals were reduced in OP50 but significantly rescued by higher MOBE concentrations. **(E)** Long reversals. were restored by 5 mg/mL MOBE, aligning closely with wild-type (N2) performance. A minimum of n = 45 biological replicates was used, consisting of three independent trials with 15 worms per group. two-way ANOVA followed by post-hoc Tukey multiple comparison test was used. Bar graphs represent the average number of responding worms across trials. Error bars indicate standard error of the mean (SEM). p < 0.05 vs. OP50.

The effects of MOBE on locomotor behaviors were examined in the UA57 *C. elegans* PD model. In untreated UA57 worms (negative controls), motor activity declined progressively over 3 days, with significant reductions in body bends (Figure 7A), total and long reversals (Figures 7D,E), and a markedly suppressed frequency of omega turns (Figure 7C). Treatment with the positive control accelerated this decline, consistent with dopamine-induced toxicity (Trist et al., 2019; Pingale and Gupta, 2021; Plotegher and Duchen, 2017).

In contrast, exposure to 5 mg/mL MOBE significantly preserved locomotor function. Body bends were maintained at near-baseline levels through Day 2, with only a mild decline observed by Day 3 (p < 0.0001 vs. negative control, Figure 7A). MOBE-treated worms exhibited a significant increase in short and total reversal events compared to both OP50 and DOPA groups (p < 0.0001, Figures 7B,D), alongside enhanced omega turn frequency and long reversals (Figures 7C,E). These findings demonstrate that MOBE-treated worms exhibited higher levels of both basic and complex motor outputs compared to controls in the UA57 PD model.

### 3.6 Phytochemical characterization of MOBE by redox-related assays

**FIGURE 8:**
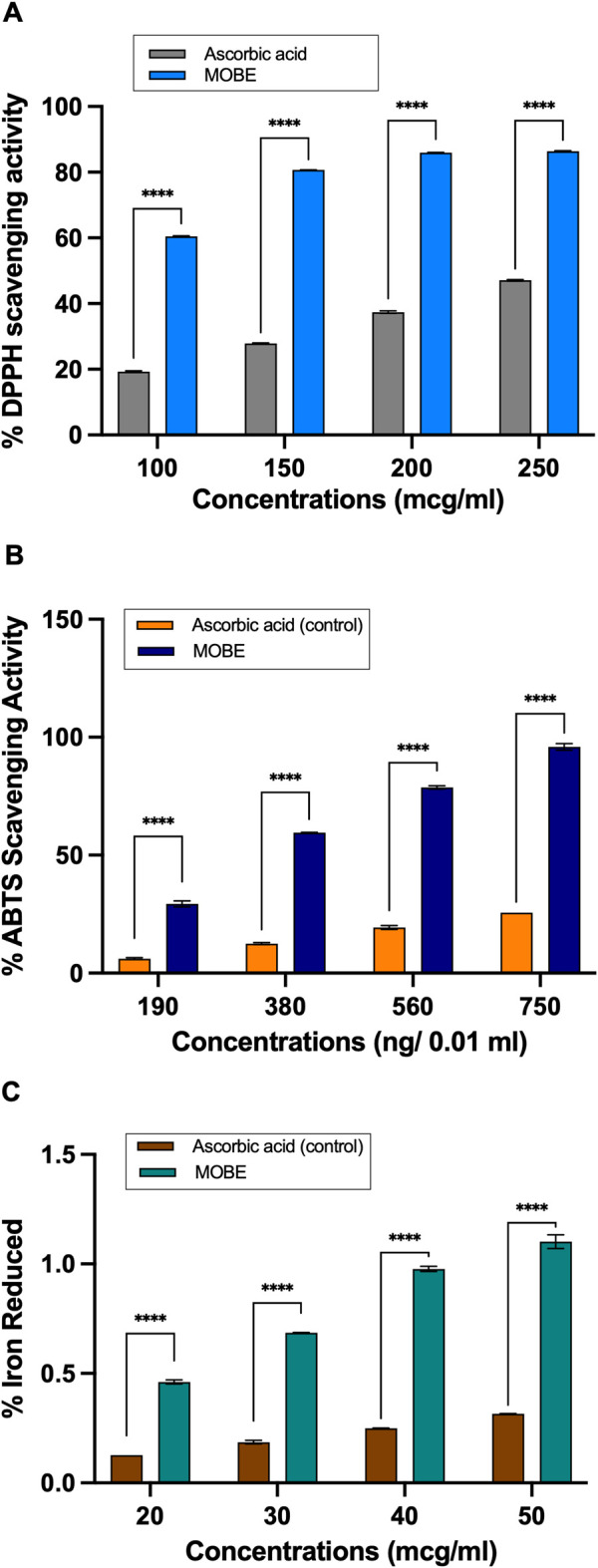
**(A)** MOBE exhibits significantly higher antioxidant activity compared to ascorbic acid in the DPPH radical scavenging assay. Ascorbic acid at concentrations of 100, 150, 200, and 250 μg/mL was used as a comparator and positive control. Data were analyzed using two-way ANOVA followed by Sidak’s multiple comparisons test. Results are presented as mean ± SD. Asterisks (***) indicate a significant difference from the control (p ≤ 0.0001). **(B)** MOBE exhibits significantly higher antioxidant activity compared to ascorbic acid in the ABTS inhibition assay. Ascorbic acid at concentrations of 190, 380, 560, and 750 ng/0.01 mL was used as the comparator and positive control. Data were analyzed using two-way ANOVA followed by Sidak’s multiple comparisons test. Results are presented as mean ± SD. Asterisks (***) indicate a significant difference from the control (p ≤ 0.0001). **(C)** MOBE exhibits significantly higher antioxidant activity compared to ascorbic acid in the Ferric Reducing Antioxidant Power (FRAP) assay. Ascorbic acid at concentrations of 20, 30, 40, and 50 μg/mL was used as the comparator and positive control. Data were analyzed using two-way ANOVA followed by Sidak’s multiple comparisons test. Results are presented as mean ± SD. Asterisks (***) indicate a significant difference from the control (p ≤ 0.0001).

The MOBE was assessed for its phytochemical profile using three chemical redox-related *in vitro* assays: DPPH radical scavenging, ABTS cation radical decolorization, and FRAP (Rumpf et al., 2023; Benzie and Strain, 1996). In the DPPH assay (Figure 8A), MOBE demonstrated radical-quenching values ranging from 32.21% to 86.38%, while in the ABTS assay (Figure 8B), the values ranged from 29.35% to 98.56%. In both cases, the measured values were higher than those observed for the ascorbic acid reference (p ≤ 0.001). In the FRAP assay (Figure 8C), MOBE exhibited reducing capacity values ranging from 29.35% to 98.56%, compared with 6.11%–31.22% for ascorbic acid (p ≤ 0.001).

### 3.7 Phenolic content and metabolite profiling of MOBE

**FIGURE 9:**
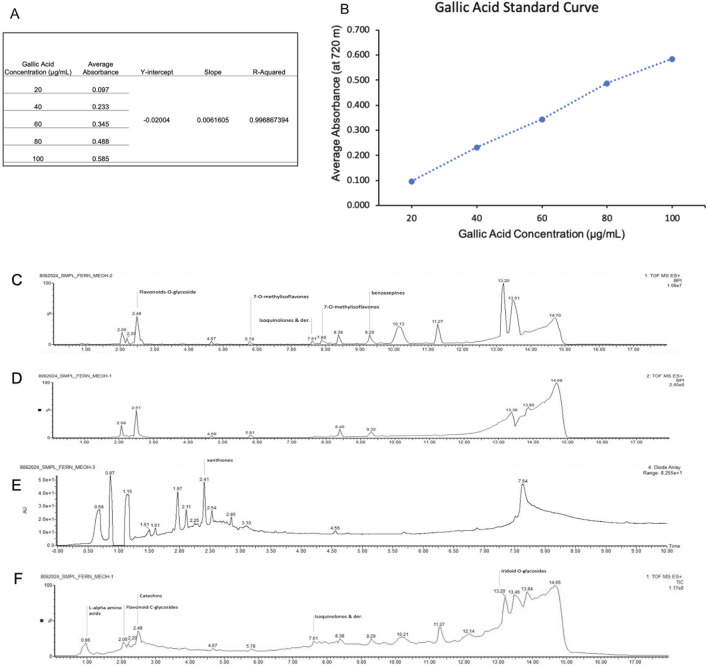
**(A,B)** Regression curve for total phenolic content of MOBE. **(C–F) (C)** MS1Chromatograms Base Peak Intensities **(D)** MS2 Chromatograms Base Peak Intensities **(E)** PDA UV Chromatograms **(F)**Total Ion Chromatograms.

To characterize MOBE at the phytochemical level, its total phenolic content and metabolite profile were analyzed. The total phenolic content, determined by the Folin–Ciocalteu method, was 22.3 mg GAE/g (Table 1), as shown in the calibration curve (Figures 9A,B). Metabolite profiling was conducted using high-resolution ultra-performance liquid chromatography coupled with electrospray ionization/quadrupole time-of-flight mass spectrometry (HR-UPLC-ESI-QTOF-MS). Data were acquired with MassLynx 4.2 software (mass range: 50–1,500 Da), converted to ABF format with Reifycs ABF Converter, and processed in MS-DIAL version 4.9 for peak detection and identification (Figure 9B). Peak annotations corresponded to the most intense ions at each retention time, representing distinct metabolites.

**TABLE 1:** *Odontosoria biflora* extract and total phenolic content.

*O. biflora* dried extract	Total phenolic content (µg GAE/g)
MOBE	22,331.76 ± 1,678.81

Eight metabolites were identified in MOBE, matched to reference libraries in MS-DIAL, with the following retention times and m/z values: 1,4-dihydroxyanthraquinone (1.720 min, 241.0501), flavonoid 8-C glycosides (2.001 min, 595.1670), 2-O-rhamnosylvitexin (2.052 min, 579.1715), khellin (2.102 min, 283.0590), isovitexin (2.178 min, 433.1134), apigenin-8-C-glucoside (2.203 min, 433.1134), benzoic acid (2.254 min, 123.0424), and pterosin G (4.235 min, 235.1337).

## 4 Discussion

This study provides the first evidence that extracts of *O. biflora* exhibit neuroprotective activity in *C. elegans* models of Parkinson’s disease. Establishing safe exposure levels was an essential first step, as plant extracts may vary considerably in toxicity depending on solvent and concentration. At 5 mg/mL, all extracts consistently maintained a survival rate of ≥90% across both wild-type and transgenic strains, validating this concentration as sublethal. DMSO was used as a solvent, and previous studies confirm that concentrations ≤0.5% exert negligible effects on worm survival, lifespan, or development (Wu et al., 2006; Alokda and Van Raamsdonk, 2022). Similar assessments in other extracts, including Impatiens balsamina and Colocasia esculenta, underscore the importance of determining safe working ranges prior to functional evaluation (Jiang et al., 2017; Bonomo et al., 2014).

Among the tested preparations, the methanolic extract (MOBE) consistently produced the most potent biological effects. In the dopaminergic neurodegeneration model UA57, which overexpresses tyrosine hydroxylase and is particularly prone to oxidative stress, MOBE preserved GFP fluorescence intensity in dopaminergic neurons. This suggests attenuation of the cascade leading from dopamine accumulation to the formation of DOPAL, dopamine quinone, and 6-hydroxydopamine, metabolites that increase ROS production and impair glutathione peroxidase activity (Plotegher and Duchen, 2017). The superior effect of MOBE is consistent with phytochemical evidence that methanol efficiently extracts phenolics, flavonoids, alkaloids, terpenoids, and tannins (Islam et al., 2021). These metabolites are recognized for their roles in antioxidant defense, anti-inflammatory regulation, and synaptic protection, thereby mitigating dopaminergic decline (Kumar et al., 2023; Rojas-García et al., 2023).

MOBE also significantly reduced the number and size of α-synuclein aggregates in the NL5901 strain, which expresses human α-synuclein fused to YFP. Since aggregate size is a more decisive determinant of toxicity than aggregate number, the observed reduction is especially relevant. Larger fibrillar aggregates are known to disrupt membranes, impair mitochondria, and induce oxidative stress (Peelaerts et al., 2015; Burre et al., 2018). These molecular effects correlated with improved locomotion and mechanosensory function, indicating that MOBE not only reduces proteotoxic stress but also preserves functional behavior *in vivo*.

Interestingly, MOBE extended lifespan in both wild-type and PD-model worms. The lack of strict correlation between aggregate reduction and lifespan extension is consistent with previous findings that aging reflects multiple overlapping processes beyond proteostasis, including oxidative damage and mitochondrial decline (Huang et al., 2019; Gallota et al., 2020; Tseng et al., 2023). This suggests that the lifespan benefits of MOBE may be mediated through broad antioxidant and stress-mitigating mechanisms.

Functional assays reinforced these observations, as MOBE improved responses to gentle head touches, plate tap tests, and locomotor patterns such as body bends, reversals, and omega turns in UA57 worms. These improvements approximated wild-type performance and were comparable to those of the positive control, supporting the notion that structural preservation of dopaminergic neurons translates into functional recovery.

Phytochemical characterization provided mechanistic support for the observed outcomes. Antioxidant assays (DPPH, FRAP, ABTS) confirmed strong radical-scavenging and reducing capacities, reflecting a high phenolic content of 22.3 mg GAE/g (Apak et al., 2013; Brand-Williams et al., 1995; Khalil et al., 2020; Noreen et al., 2017). Metabolite profiling revealed the presence of bioactive metabolites including 1,4-dihydroxyanthraquinone, flavonoid 8-C glycosides such as isovitexin and 2-O-rhamnosylvitexin, apigenin-8-C-glucoside, khellin, benzoic acid, and pterosin G. These metabolites are associated with neuroprotection in diverse models: anthraquinones modulate antioxidant enzymes (Zhang et al., 2021), flavonoids inhibit NF-κB signaling and scavenge ROS (Kurutas, 2015; Barreca et al., 2023; Martínez-Coria et al., 2023), apigenin-8-C-glucoside protects dopaminergic neurons by regulating Bax/Bcl-2 pathways (Mushtaq et al., 2023; Salehi et al., 2019), benzoic acid reduces lipid peroxidation while preserving dopamine metabolism (Zaidi et al., 2023), and pterosin G contributes additional antioxidant activity (Baskaran et al., 2018). The co-occurrence of these metabolites suggests a synergistic effect, whereby multiple compounds act on convergent yet distinct pathways to maintain neuronal health.

The translational value of these findings lies in the multi-level convergence of molecular, cellular, and behavioral protection. MOBE reduced dopaminergic neuronal loss, suppressed α-synuclein aggregation, improved motor and sensory behavior, and extended lifespan, outcomes highly relevant to the multifactorial pathology of PD (Shoaib et al., 2023). Such broad-spectrum effects highlight the therapeutic promise of complex botanical extracts compared with single-target drugs.

Nevertheless, this work has limitations. The assays employed a single concentration in some models, limiting EC_50_ estimation and full characterization of the therapeutic window. α-Synuclein expression in worm muscle cells does not fully replicate the pathology of Lewy bodies in mammalian neurons, thereby limiting translational accuracy. Furthermore, while metabolite profiling identified candidate metabolites, the specific contribution of each metabolite remains unresolved. Future work should incorporate dose–response analyses, metabolite fractionation, mechanistic validation, and mammalian studies to establish translational potential.

## 5 Conclusion

In conclusion, methanolic extracts of *O. biflora* exhibit multi-dimensional neuroprotective activity in *C. elegans* PD models, mediated at least in part by phenolic and flavonoid metabolites with potent antioxidant and anti-inflammatory properties. By reducing dopaminergic neuronal loss, attenuating α-synuclein aggregation, extending lifespan, and restoring behavioral function, MOBE demonstrates promise as a botanical drug candidate. Further systematic and translational studies are warranted to confirm these effects and advance *O. biflora* toward therapeutic development.

## Data Availability

The original contributions presented in the study are included in the article/supplementary material, further inquiries can be directed to the corresponding author.
